# Quality and Dissemination of Uterine Fibroid Health Information on TikTok and Bilibili: Cross-Sectional Study

**DOI:** 10.2196/75120

**Published:** 2025-08-01

**Authors:** Lan Wang, Yiwen Chen, Duo Zhao, Tao Xu, Fu Hua

**Affiliations:** 1 Department of Obstetrics and Gynecology, Huai'an 82 Hospital, Huai'an, China; 2 Department of Endocrinology, Nanjing University of Chinese Medicine, Nanjing Drum Tower Hospital, Nanjing, China; 3 Department of Orthopedics, The First People's Hospital of Guannan: Lianyun, Lianyungang, China; 4 Department of Gynecology, Nanjing Medical University, Huaian First People’s Hospital, No. 1 West Huanghe Road, Huaiyin District, Huai'an, 223300, China, 86 15905234818

**Keywords:** uterine fibroids, videos quality, TikTok, Bilibili, modified DISCERN, PEMAT-A/V, Spearman correlation analysis, stepwise regression analysis, Patient Education Materials Assessment Tool-Audiovisual Materials, Global Quality Score

## Abstract

**Background:**

The rise of short-video platforms, such as TikTok (Douyin in China) and Bilibili, has significantly influenced how health information is disseminated to the public. However, the quality, reliability, and effectiveness of health-related content on these platforms, particularly regarding uterine fibroids, remain underexplored. Uterine fibroids are a common medical condition that affects a substantial proportion of women worldwide. While these platforms have become vital sources of health education, misinformation and incomplete content may undermine their efficacy.

**Objective:**

This study aims to address these gaps by evaluating the quality and dissemination effectiveness of uterine fibroid–related health information on TikTok and Bilibili.

**Methods:**

A total of 200 uterine fibroid-related videos (100 from TikTok and 100 from Bilibili) were selected through a keyword search. The videos were evaluated by 2 trained gynecological experts using the Global Quality Score (GQS) and a modified DISCERN (mDISCERN) tool. In addition, the Patient Education Materials Assessment Tool for Audio and Visual Materials was used to assess the understandability and actionability of the videos. Statistical analyses, including the Mann-Whitney *U* test, Spearman rank correlation, and stepwise regression analysis, were used to assess differences between platforms and identify predictors of video quality.

**Results:**

The results indicated that TikTok outperformed Bilibili in terms of user engagement metrics, such as likes, comments, shares, and followers (all *P*<.001). However, Bilibili videos were generally longer than those on TikTok (*P*<.001). The videos on both platforms demonstrated suboptimal overall quality and reliability, reflected by median GQS score of 3 (IQR 3-4) for TikTok and the median GQS score of Bilibili is 3 (IQR 2-4). The median modified DISCERN scores were also low: 2 (IQR 2‐2) for TikTok and 2 (IQR 2‐2) for Bilibili, with no significant differences between the 2 platforms (*P*=.62 for GQS; *P*=.18 for mDISCERN). The videos on both platforms yielded comparable median scores for Patient Education Materials Assessment Tool-Understandability (PEMAT-U) and Patient Education Materials Assessment Tool-Actionability (PEMAT-A). The median score of PEMAT-U was 77% (IQR 69%-83%) for TikTok and 77% (IQR 69%-85%) for Bilibili. The PEMAT-A yielded a median score of 67% (IQR 33%-67%) for TikTok and 67% (IQR 0-67%) for Bilibili. Videos uploaded by medical professionals on TikTok had significantly higher quality scores compared to those uploaded by nonprofessionals. A moderate positive correlation was observed between the GQS and mDISCERN scores (*r*=0.41, *P*<.01), indicating an interrelationship between quality and reliability. Stepwise regression analysis identified “completeness score,” “source,” and “PEMAT scores” as significant predictors of video quality.

**Conclusions:**

This study highlights the generally low quality of uterine fibroid–related health information on short-video platforms, although TikTok showed better performance in terms of engagement and quality. The involvement of medical professionals was found to enhance video quality. These findings underscore the need for improved oversight of health content on social media platforms and greater involvement of health care professionals to ensure the dissemination of accurate and reliable health information.

## Introduction

Uterine fibroids are the most prevalent benign tumors of the female reproductive system, affecting approximately 70% of women of reproductive age worldwide, particularly women of color, with an increasing incidence rate [1]. Approximately 30% of uterine fibroids manifest clinical symptoms, predominantly including abnormal uterine bleeding, compression symptoms affecting surrounding organs and tissues (such as constipation and urinary frequency), pelvic pain, and infertility [2]. Furthermore, uterine fibroids exert a significant impact on women’s reproductive health, influencing fertility potential and pregnancy outcomes [3]. Primary risk factors for the condition include age, race, endogenous and exogenous hormonal factors, obesity, uterine infections, and lifestyle factors (including diet, caffeine and alcohol consumption, physical activity, stress, and smoking) [4]. Ultrasound is the preferred imaging modality, supplemented by magnetic resonance imaging when necessary [5]. Principal treatment modalities include pharmacological therapy, surgical intervention, and interventional procedures; surgical options comprise hysterectomy, myomectomy, uterine artery embolization, and focused ultrasound surgery [5]. Individualized and long-term management is paramount.

With the burgeoning ubiquity of the internet and the expanding reach of digital information, the internet has become a prevalent public resource for acquiring health knowledge and investigating personal health conditions [6]. As early as 2005, research indicated that the internet constituted one of the primary sources for adolescents seeking health information [7]. With advancements in technology, short videos centered on patients’ diseases have been used in medical education, garnering positive evaluations [8]. Online short videos, as an emerging form of video media, have achieved substantial dissemination reach [9]. The public and health care professionals can engage in dialogue on health issues via social media, potentially enhancing health outcomes [10]. However, misinformation and disinformation on social media amplify the risks inherent in digital health communication, posing a challenge to the efficacy of public health measures [11].

Short-video platforms, such as TikTok/Douyin and Bilibili, have become significant channels through which the public accesses health information. While platforms like TikTok/Douyin (the international version of Douyin) and Bilibili have achieved immense popularity and reach [9,12], their role in disseminating health content necessitates a critical examination of the quality of this information. Understanding how users interact with and evaluate health content on these platforms is crucial, particularly in light of concepts such as digital health literacy and the potential for misinformation dissemination.

Prior studies have investigated the quality of health–related videos on these platforms concerning various conditions, such as adolescent vision health [13], lung cancer [14], liver cancer [15], brain tumor [16], breast cancer [17], heart failure [18], thyroid nodules [19], gallstone disease [20], chronic obstructive pulmonary disease [21], etc. Topics related to the female reproductive system, including oral contraceptives [22], contraception-related videos [23], and cervical cancer [24], have also been subjects of research. However, studies specifically focusing on the particular condition of uterine fibroids remain notably scarce. Given that misinformation in the field of reproductive health can adversely affect health outcomes, weaken public trust in medical systems, and potentially lead to inappropriate policy restrictions [25], evaluating the quality of information regarding uterine fibroids on these high-traffic platforms is of pressing importance.

While short-video platforms have rapidly become significant conduits for the public to access gynecological health knowledge, a critical gap exists in the systematic evaluation of the quality and reliability of content specifically pertaining to uterine fibroids on these platforms. Addressing this crucial research gap, this study aims to rigorously assess the quality, reliability, and dissemination characteristics of health information on uterine fibroids across TikTok and Bilibili. By analyzing differences between platforms and publisher types, this research seeks to provide a scientific foundation for improving the effective and accurate dissemination of uterine fibroids–related health information, thereby contributing to better public health literacy and outcomes in this area.

## Methods

### Data Collection and Screening

**Figure 1.**
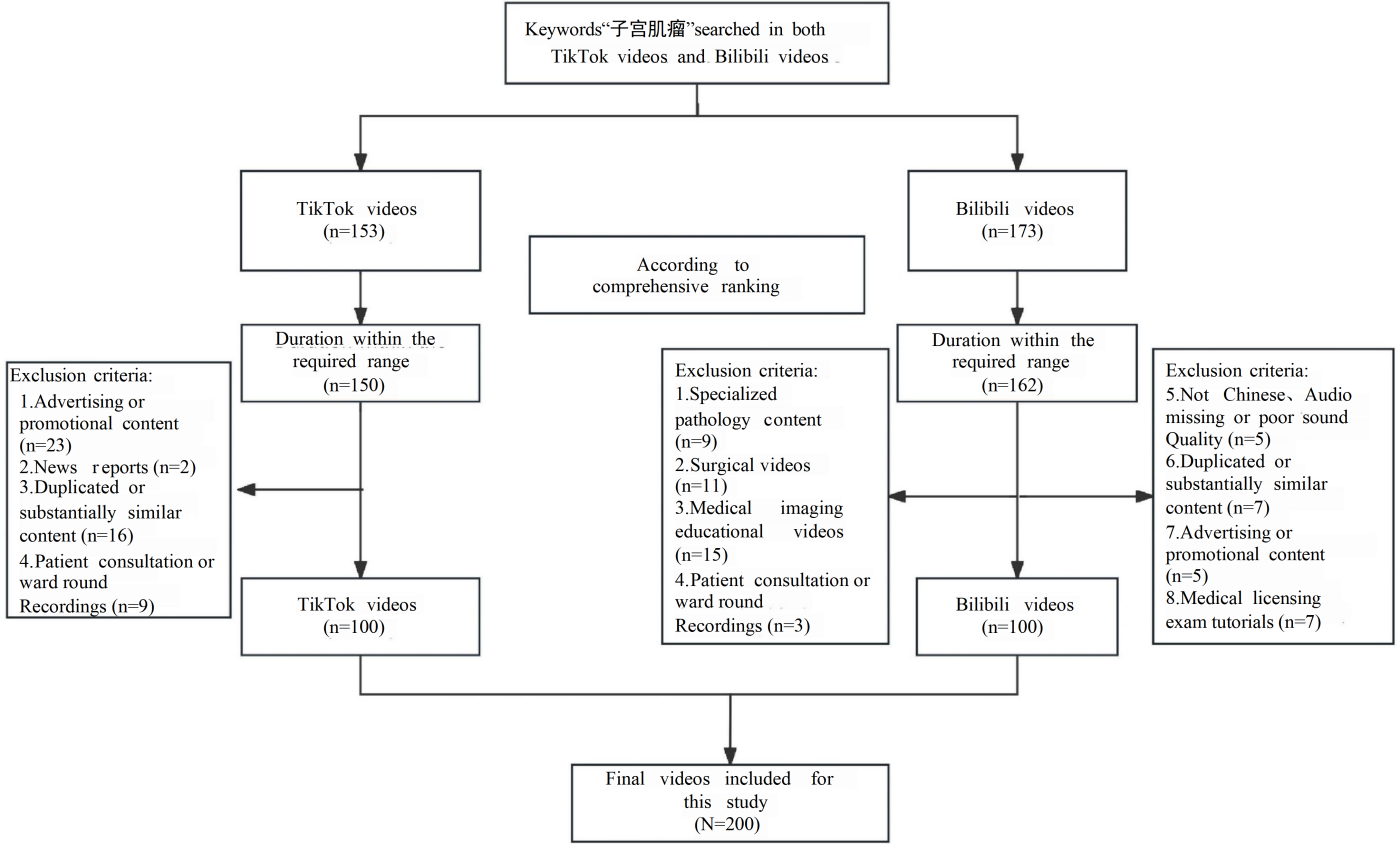
Flowchart for searching short videos on uterine fibroids (March 3‐7, 2025; cross-sectional study).

In this cross-sectional study, new accounts were registered on the TikTok and Bilibili platforms between March 3 and March 7, 2025. Video searches were conducted using the Chinese keyword “子宫肌瘤" (uterine fibroids), and the top 100 comprehensively ranked relevant videos from each platform were selected (Figure 1). Inclusion criteria included videos addressing uterine fibroids, presented in Chinese, with durations ranging from 20 seconds to 15 minutes. Exclusion criteria encompassed videos unrelated to uterine fibroids, duplicates or substantially similar videos, promotional advertisements, news reports, surgical or pathology-specific videos, outpatient consultation or ward round recordings, medical exam instructional videos, non–Chinese-language videos, videos with missing audio or poor sound quality, and those uploaded by users with unverified identities. Ultimately, 200 videos were included (100 from TikTok and 100 from Bilibili). The analysis was restricted to the top 100 videos per platform, as prior studies have demonstrated that videos beyond this threshold exert no significant influence on the analysis [15,26].

### Data Collection Content

The uploader’s identity, follower count, video duration, number of likes, comments, favorites, and shares were recorded. All extracted data were documented in Excel (Microsoft Corp). The dataset is available in Multimedia Appendix 1.

Platforms were designated as 1=TikTok and 2=Bilibili. Videos were categorized into 4 groups based on source and 5 groups based on content. Video sources were classified as follows: (1) professional individuals, including gynecologists, radiologists, and oncologists; (2) nonprofessional individuals, encompassing practitioners of traditional Chinese medicine, physicians from other disciplines, individuals with medical backgrounds, and those without medical backgrounds; (3) professional institutions; and (4) nonprofessional institutions. Video content was classified by theme as follows: (1) disease-related knowledge, (2) etiology, (3) symptoms, (4) treatment, and (5) prevention and prognosis.

#### Quality Assessment Tools

#### Global Quality Score

Ranging from 1 to 5 points, with higher scores indicating superior quality [27], this metric evaluates accuracy, authority, completeness, and educational value (Table S1 in Multimedia Appendix 2).

#### Modified DISCERN Tool

Comprising 5 questions adapted from the original DISCERN tool, it is used to assess the reliability of health information [28], evaluating clarity, relevance, traceability, scientific basis, and impartiality. Each dimension is scored 1 point for “yes” and 0 points for “no” (Textbox 1).

Textbox 1. **Modified DISCERN quality criteria for assessing the reliability of video. (1 point for answer “yes,” 0 point for answer “no”).**

**Reliability score**

- Is the video clear, concise, and understandable?
- Are valid sources cited?
- Is the content presented balanced and unbiased?
- Are additional sources of content listed for patient reference?
- Are areas of uncertainty mentioned?

#### The Patient Education Materials Assessment Tool

Using the Patient Education Materials Assessment Tool (PEMAT) [29] for audiovisual materials (PEMAT-A/V) with an automated scoring form, which assesses the understandability and actionability of videos.

### Statistical Analysis

Data analysis was performed using SPSS version 27.0 (IBM Corp). Descriptive statistics were presented as median (IQR). The Mann-Whitney *U* test was used to compare quality differences between platforms (TikTok vs Bilibili), publisher, and across video content categories. Spearman’s rank correlation coefficient (ρ) and stepwise regression analysis were calculated to evaluate the association between quality scores and engagement metrics (likes, shares, and comments). The Cohen κ coefficient was used to assess interrater reliability among experts. Statistical significance was set at *P*<.05 for all tests.

### Ethical Considerations

This study did not use clinical data, human specimens, or laboratory animals. All information was derived from publicly released videos on TikTok and Bilibili platforms, with no data involving personal privacy issues. Furthermore, this research did not involve any interaction with users; therefore, ethical review was not required.

## Results

### Videos Characteristics

The overall characteristics of the videos are summarized in Table 1. TikTok videos exhibit significantly higher numbers of likes, comments, favorites, shares, and publisher follower counts compared to Bilibili videos (all *P*<.001), whereas Bilibili videos are notably longer in duration than TikTok videos (*P*<.001).

**Table 1.** Characteristics of uterine fibroid videos on TikTok and Bilibili.

Variable	TikTok (n=100), median (IQR)	Bilibili (n=100), median (IQR)	Wilcoxon rank-sum test	
			Z score	*P* value
Likes	431 (249‐1238)	44 (9‐267)	−7.68	<.001
Comments	35 (12‐93)	4 (0‐28)	−6.5	<.001
Collections	129 (67‐437)	30 (4‐150)	−5.71	<.001
Shares	72 (32‐220)	14 (2‐108)	−4.95	<.001
Duration	66 (46‐91)	138 (80‐294)	−7.2	<.001
Followers	115,000 (27,750‐511,750)	14,000 (1212‐65,000)	−6.76	<.001
GQS^a^	3 (3‐4)	3 (2‐4)	−0.49	.62
mDIS^b^	2 (2‐2)	2 (2‐2)	−1.35	.18
PEMAT-U^c^, %	77 (69‐83)	77 (69‐85)	−0.72	.47
PEMAT-A^d^, %	67 (33‐67)	67 (0‐67)	−0.29	.77

a GQS: Global Quality Score.

b mDIS: modified DISCERN.

c PEMAT-U: Patient Education Materials Assessment Tool-Understandability score.

d PEMAT-A: Patient Education Materials Assessment Tool-Actionability score.

The median Global Quality Score (GQS) for TikTok videos is 3 (IQR 3‐4), with a median modified DISCERN (mDISCERN) score of 2 (IQR 2‐2). For Bilibili videos, the median GQS and mDISCERN scores are 3 (IQR 2‐4) and 2 (IQR 2‐2), showing no statistically significant differences between the 2 platforms (*P*=.62 for GQS and *P*=.18 for mDISCERN), respectively. Interrater agreement between the 2 evaluators was quantified using Cohen κ. According to the criteria proposed by Landis and Koch [30], a κ value >0.8 indicates excellent agreement, values between 0.6 and 0.8 denote substantial agreement, values between 0.4 and 0.6 reflect moderate agreement, and values <0.4 suggest poor agreement. The Cohen κ values for GQS and mDISCERN scores were 0.727 and 0.725, indicating substantial interrater agreement, respectively.

The median PEMAT-U (understandability) score for TikTok videos is 77% (IQR 69%‐83%), with a median PEMAT-A (actionability) score of 67% (IQR 33%‐67%). For Bilibili videos, the median PEMAT-U and PEMAT-A scores are 77% (IQR 69%‐85%) and 67% (IQR 0%‐67%), with no statistically significant differences between the platforms (*P*=.47 for PEMAT-U and *P*=.77 for PEMAT-A), respectively. Tables S2-S4 in Multimedia Appendix 2 along with Table 2, illustrate the sources and content types of videos on TikTok and Bilibili.

**Table 2.** Percentage distribution of uterine fibroid videos by source content and category on TikTok and Bilibili.

		Distribution of uterine fibroid videos, %
Sources of TikTok videos		
	Nonprofessional individuals	1
	Nonprofessional institutions	0
	Professional individuals	83
	Professional institutions	16
Sources of Bilibili videos		
	Nonprofessional individuals	6
	Nonprofessional institutions	3
	Professional individuals	52
	Professional institutions	39
Content categories of TikTok videos		
	Disease knowledge	31
	Etiology	5
	Prevention and prognosis	11
	Symptoms	5
	Treatment	48
Content categories of Bilibili videos		
	Disease knowledge	32
	Etiology	3
	Prevention and prognosis	30
	Symptoms	4
	Treatment	31

On TikTok, professional individuals uploaded the majority of videos, accounting for 83% (83/100), a significantly high proportion, followed by nonprofessional individuals (16/100, 16%) and professional institutions (1/100, 1%). Regarding video content on TikTok, the majority focused on treatment (48/100, 48%), followed by disease knowledge (31/100, 31%), prevention and prognosis (11/100, 11%), etiology (5/100, 5%), and symptoms (5/100, 5%). On Bilibili, professional individuals accounted for 52% of video sources (52/100), a proportion significantly lower than on TikTok, while non-professional individuals comprised 39% (39/100). This disparity is closely linked to the publisher verification policies of the two platforms: TikTok enforces stricter credential verification for medical personnel and imposes rigorous restrictions on medical advertisements and pharmaceutical promotions. On Bilibili, content pertaining to disease knowledge (32%), treatment (31%), and prevention and prognosis (30%) is represented in roughly equal proportions.

### Video Content Distribution

**Figure 2.**
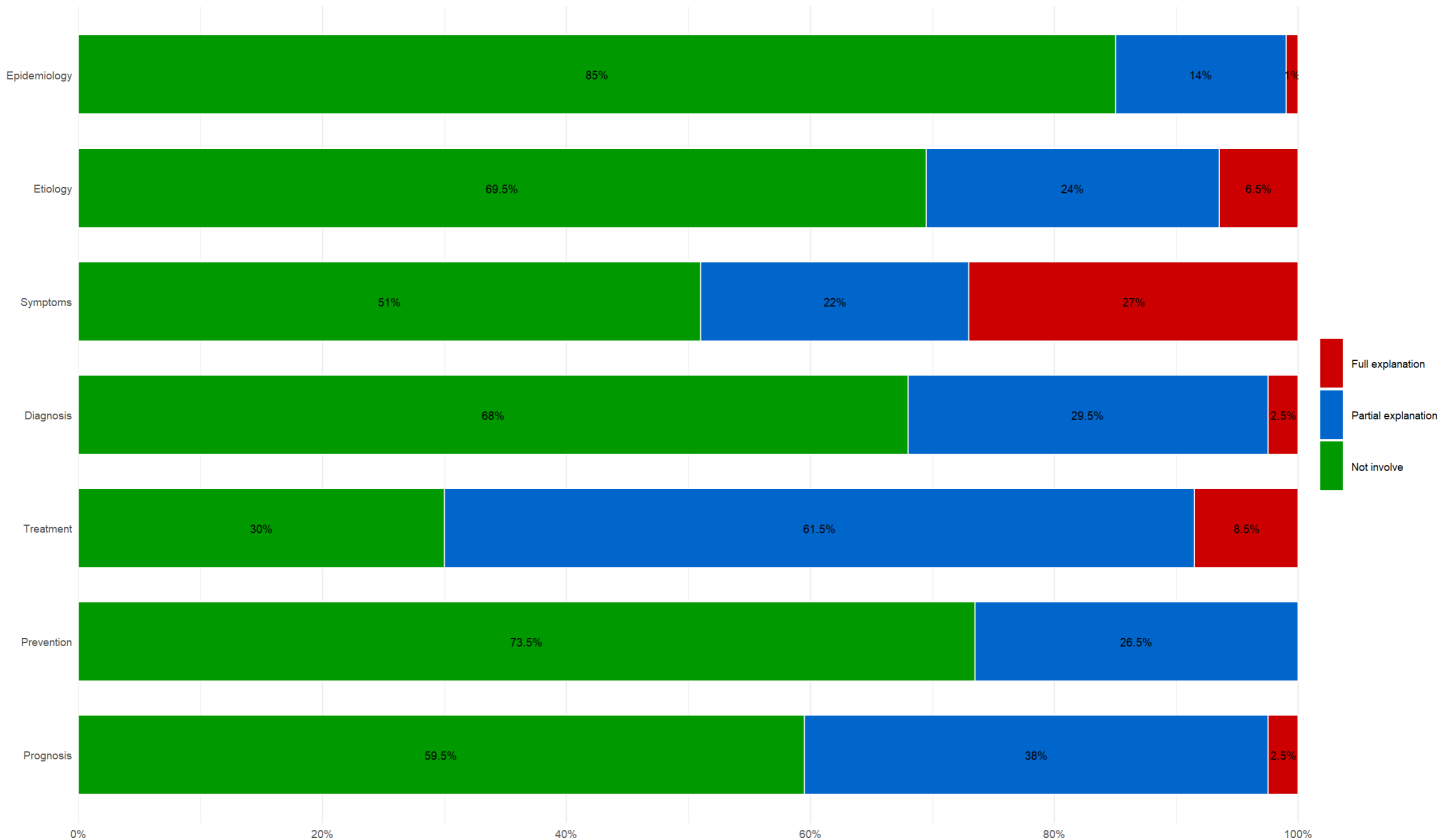
Percentage distribution of explanatory coverage across different domains in uterine fibroid video content.

We further analyzed the content discussed in the included short videos (Table S5 in Multimedia Appendix 2) and evaluated their completeness (Multimedia Appendix 3) across 7 domains: epidemiology, etiology, symptoms, diagnosis, treatment, prevention, and prognosis. Each domain was scored from 0 to 2 points, with a total possible score of 14 points. A subdomain mentioned once received 1 point (partial explanation), more than 3 mentions received 2 points (complete explanation), and no mention received 0 points. The scores for each domain are presented in Figure 2. We found that the treatment of uterine fibroids was the most frequently discussed topic, addressed in 70% (140/200) of the total 200 videos (123/200, 61.5% partial explanation and 17/200, 8.5% complete explanation). On TikTok, 77% (77/100) of videos provided partial or full explanations of treatment, compared to 63% (63/100) on Bilibili. Symptoms followed as the second most common topic, appearing in 49% (98/200) of the total videos. Etiology and diagnosis of uterine fibroids also garnered relative public interest, with similar proportions (30.5% vs 32%). However, few videos offered detailed health information on the epidemiology of uterine fibroids, with only 12% (12/100) of TikTok videos providing partial (11/100, 11%) or full (1/100, 1%) explanations. Notably, no videos provided a detailed explanation of prevention strategies for uterine fibroids.

**Figure 3.**
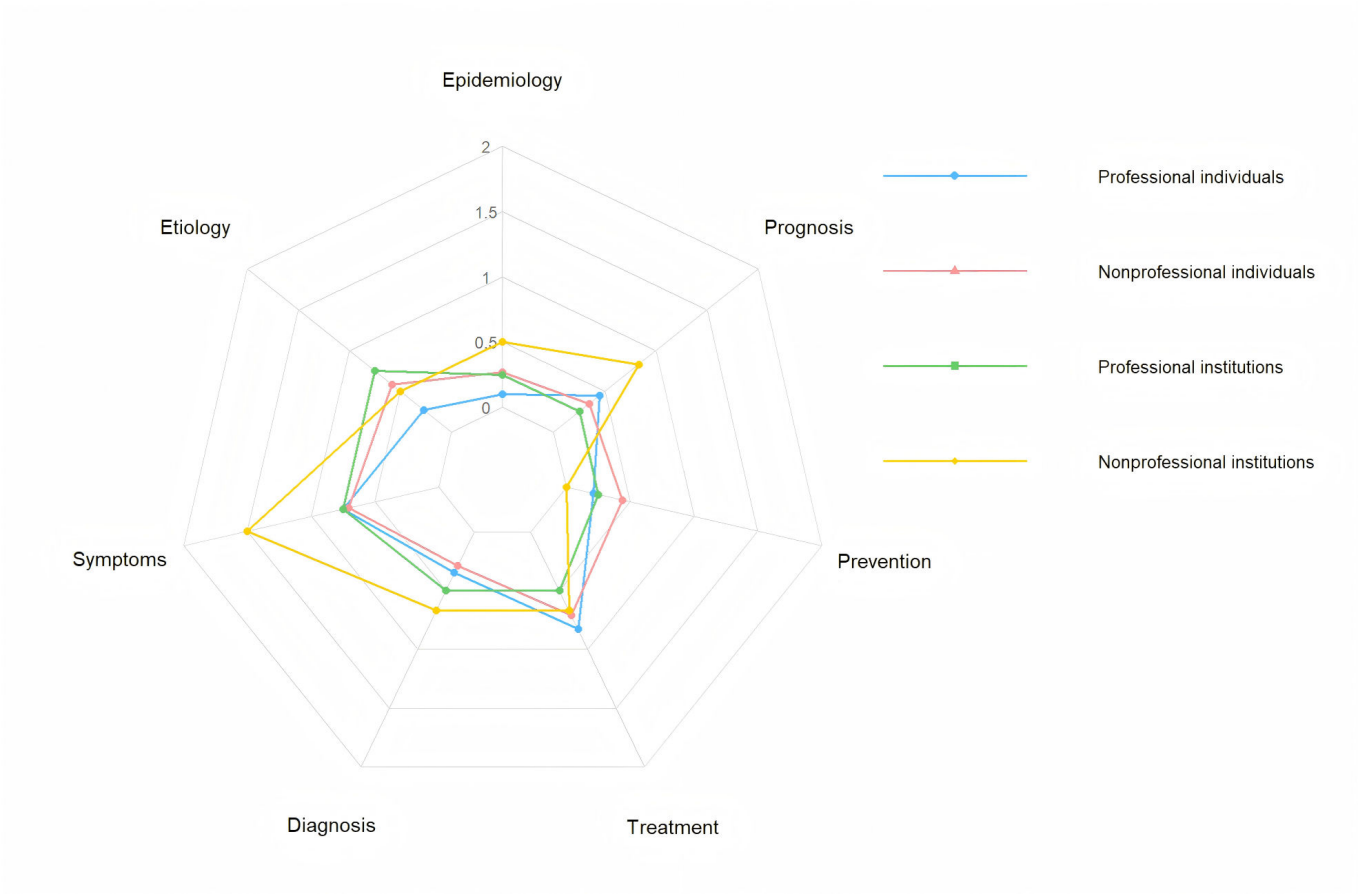
Multidimensional content quality assessment of uterine fibroid videos using radar charts, stratified by source.

**Figure 4.**
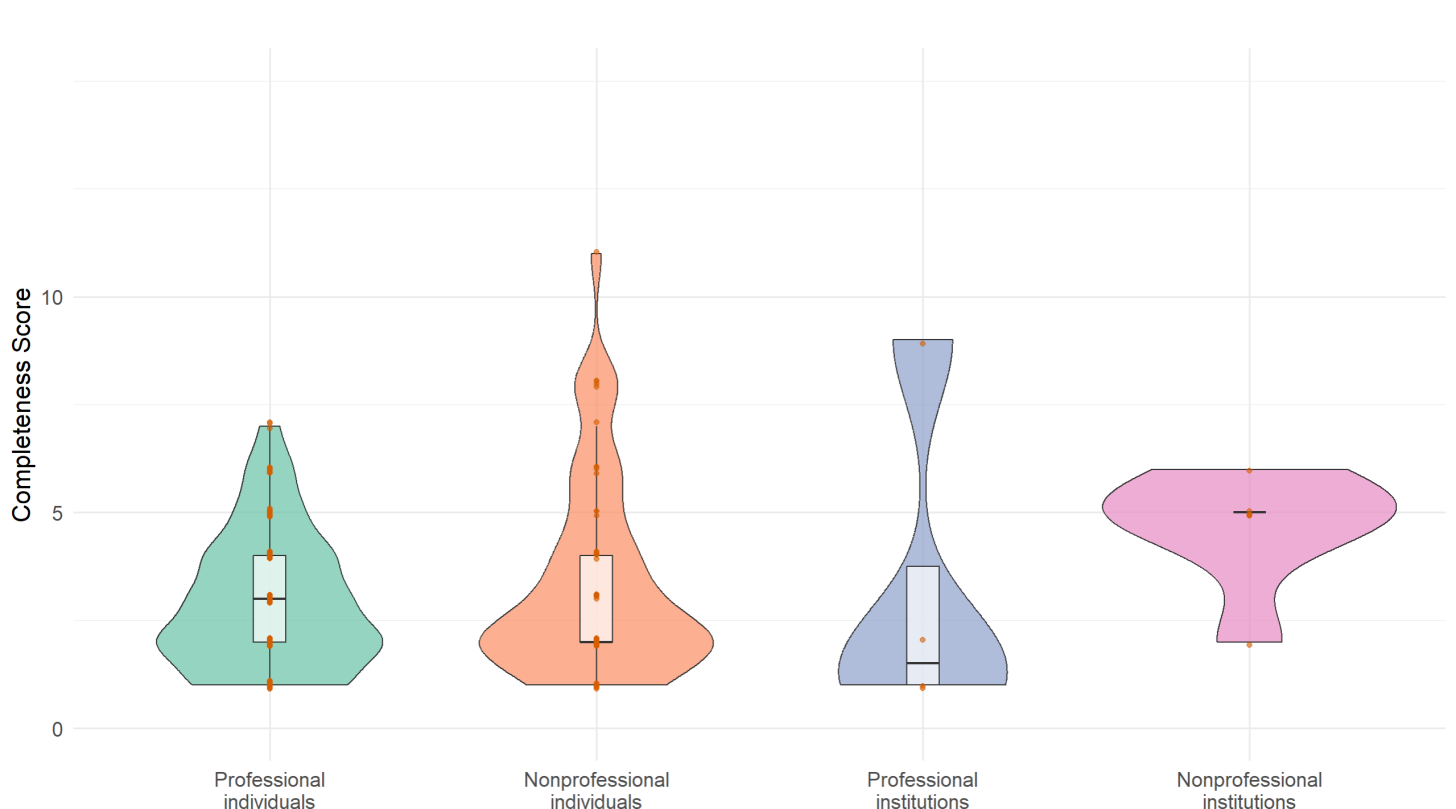
Probability density distribution comparison of content integrity composite scores for uterine fibroid videos.

Videos covered the 7 predefined content domains of the completeness scoring to varying degrees (Table S6 in Multimedia Appendix 2, Figure 3). Nonprofessional institutions scored highest in the “symptoms” (mean 1.50, SD 0.84) and “prognosis” (mean 0.83, SD 0.41) domains but recorded a score of 0 in the “prevention” domain, indicating a complete absence of prevention-related information from this source. Professional individuals performed well in the “treatment” (mean 0.83, SD 0.58) and “symptoms” (mean 0.75, SD 0.86) domains but scored lowest in “epidemiology” (mean 0.10 SD 0.32), suggesting limited coverage in this area. Professional institutions achieved higher scores in the “etiology” and “symptoms” domains (mean 0.75, SD 0.96) but scored lower in “prevention” and “prognosis” (mean 0.25, SD 0.50), possibly due to a focus on academic rigor at the expense of practical utility. The mean score for “symptoms” from non-professional institutions (1.50, SD 0.84) was significantly higher than that of other sources, likely because their content aligns more closely with public needs. Based on Figure 4, professional institutions emerge as the most reliable sources for comprehensive information, demonstrating broad coverage across various disease topics coupled with higher completeness. Conversely, information originating from nonprofessional individuals exhibits notable limitations in overall comprehensiveness. The radar plot reveals weaker coverage in several critical domains, including epidemiology, diagnosis, and prognosis, while the violin plot indicates a wider distribution of their completeness scores. While the radar chart emphasizes the breadth of topics addressed, the violin plot focuses on the completeness score. The congruence and discrepancies between these visualizations provide valuable insights into the strengths and weaknesses of diverse information sources.

### Video Quality and Reliability Assessments

**Figure 5.**
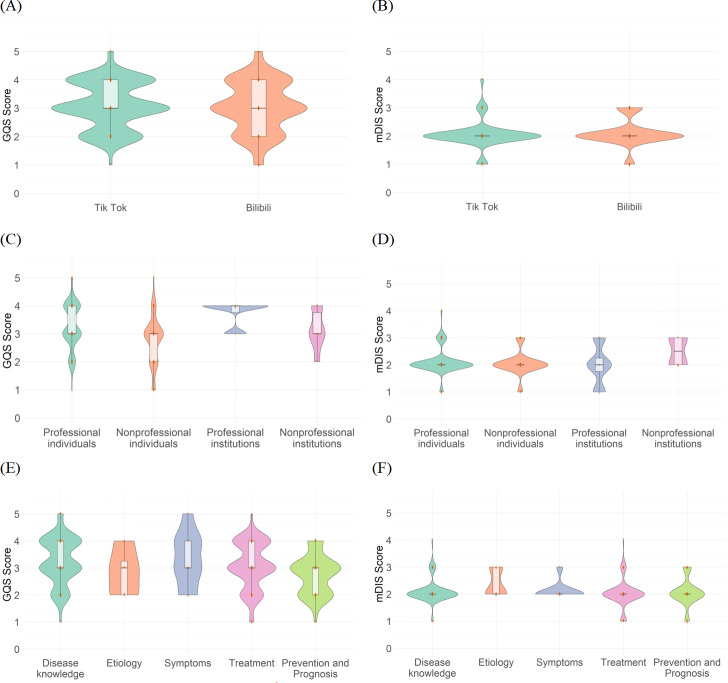
Global Quality Score and modified DISCERN for Uterine Fibroid Videos. (A) Comparison of Global Quality Scores between TikTok and Bilibili videos. (B) Comparison of modified DISCERN scores between TikTok and Bilibili videos. (C) Global Quality Scores across different video sources. (D) Modified DISCERN scores across different video sources. (E) Global Quality Scores across different content categories. (F) Modified DISCERN scores across different content categories.GQS: Global Quality Score; mDIS: modified DISCERN.

The GQS and mDISCERN scores were compared across different platforms, video sources, and content types. The GQS scores for TikTok and Bilibili (Figure 5A) are predominantly clustered around 3, indicating moderate video quality. Although Bilibili videos exhibit a concentration of higher scores, a greater number of videos are clustered at the lower end, which drags down the overall quality level. The mDISCERN scores (Figure 5B) reveal moderate reliability for both platforms. There is no significant difference in quality and reliability between the 2 platforms, with TikTok slightly outperforming Bilibili.

Regarding video sources, GQS scores for professional individuals and professional institutions show a concentration of higher scores, reflecting superior quality. Nonprofessional institutions, however, achieve a median GQS score of 4 (Figure 5C), significantly higher than other groups (professional groups and nonprofessional individuals, both at 3), and a median mDISCERN score of 3 (Figure 5D), surpassing the median of 2 for other groups. This suggests that content from nonprofessional institutions excels in both quality and reliability, challenging the traditional assumption that professional sources consistently dominate. However, this finding is tempered by the limited number of videos from nonprofessional institutions, rendering it unrepresentative of the broader dataset. The score distribution for nonprofessional individuals displays considerable variability, underscoring the heterogeneity in content quality and reliability within this group.

In terms of content domains, the GQS scores for disease knowledge (Figure 5E) exhibit a concentration of high scores with low dispersion, indicating high quality and strong information authority. Similarly, in mDISCERN scores (Figure 5F), disease knowledge demonstrates greater reliability, outperforming other categories.

### Video Understandability and Actionability

**Figure 6.**
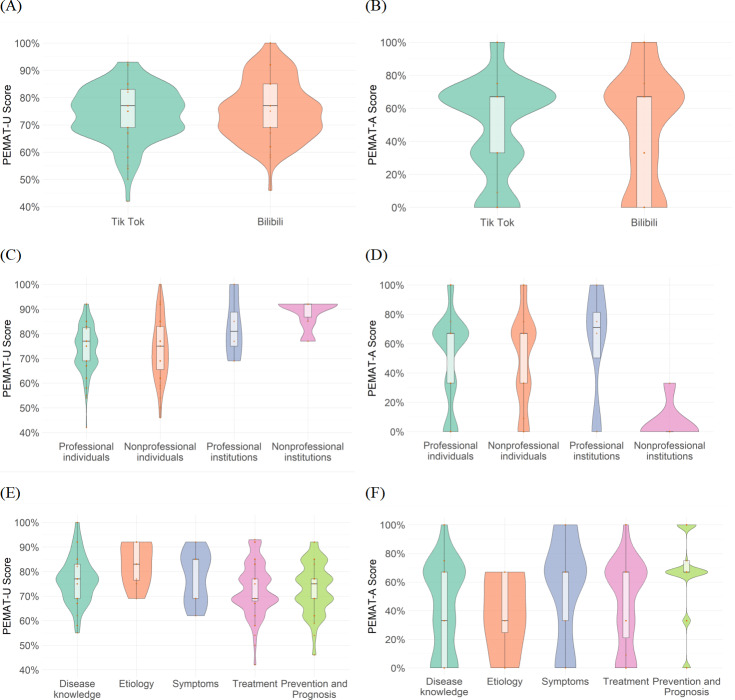
Comparative analysis of video understandability (PEMAT-U) and Actionability (PEMAT-A) of Uterine Fibroid Videos. (A) Patient Education Materials Assessment Tool-Understandability scores of videos across TikTok and Bilibili. (**B**) Patient Education Materials Assessment Tool-Actionability scores of videos across TikTok and Bilibili. (**C**) Patient Education Materials Assessment Tool-Understandability scores of videos across different sources. (**D**) Patient Education Materials Assessment Tool-Actionability scores of videos across different sources. (**E**) Patient Education Materials Assessment Tool-Understandability scores of videos across different content categories. (**F**) Patient Education Materials Assessment Tool-Actionability scores of videos across different content categories. PEMAT-A: Patient Education Materials Assessment Tool-Actionability. PEMAT-U: Patient Education Materials Assessment Tool-Understandability.

A comparison of Patient Education Materials Assessment Tool for Understandability (PEMAT-U) scores across video sources on different platforms (Figure 6A) reveals that TikTok videos exhibit a concentration of higher scores, outperforming Bilibili in understandability. Although the highest understandability score was derived from Bilibili. This, combined with the shorter duration of TikTok videos, suggests that their content is concise, with strong visual aids. However, lower scores stem from oversimplification and insufficient information completeness. In terms of PEMAT-A (actionability) scores (Figure 6B), TikTok similarly surpasses Bilibili.

Regarding understandability across video sources, nonprofessional individuals demonstrate a stronger advantage (Figure 6C), likely due to their use of more colloquial language, while professional individuals show significant variation; the scores ranged from a minimum of 42% to a maximum of 100%. For actionability, professional individuals, nonprofessional individuals, and professional institutions all have a median score of 67%, with no notable differences; however, professional institutions exhibit marked polarization. In contrast, videos from nonprofessional institutions consistently show lower actionability, indicating poor feasibility (Figure 6D).

Concerning video content, the “symptoms” domain achieves the highest understandability while the “prevention and prognosis” domain scores lower (Figure 6E). Overall, no understandability scores fall below 42%, whereas actionability scores include a notable proportion of low values, even reaching 0% (Figure 6F).

### Correlation and Stepwise Regression Analysis

**Figure 7.**
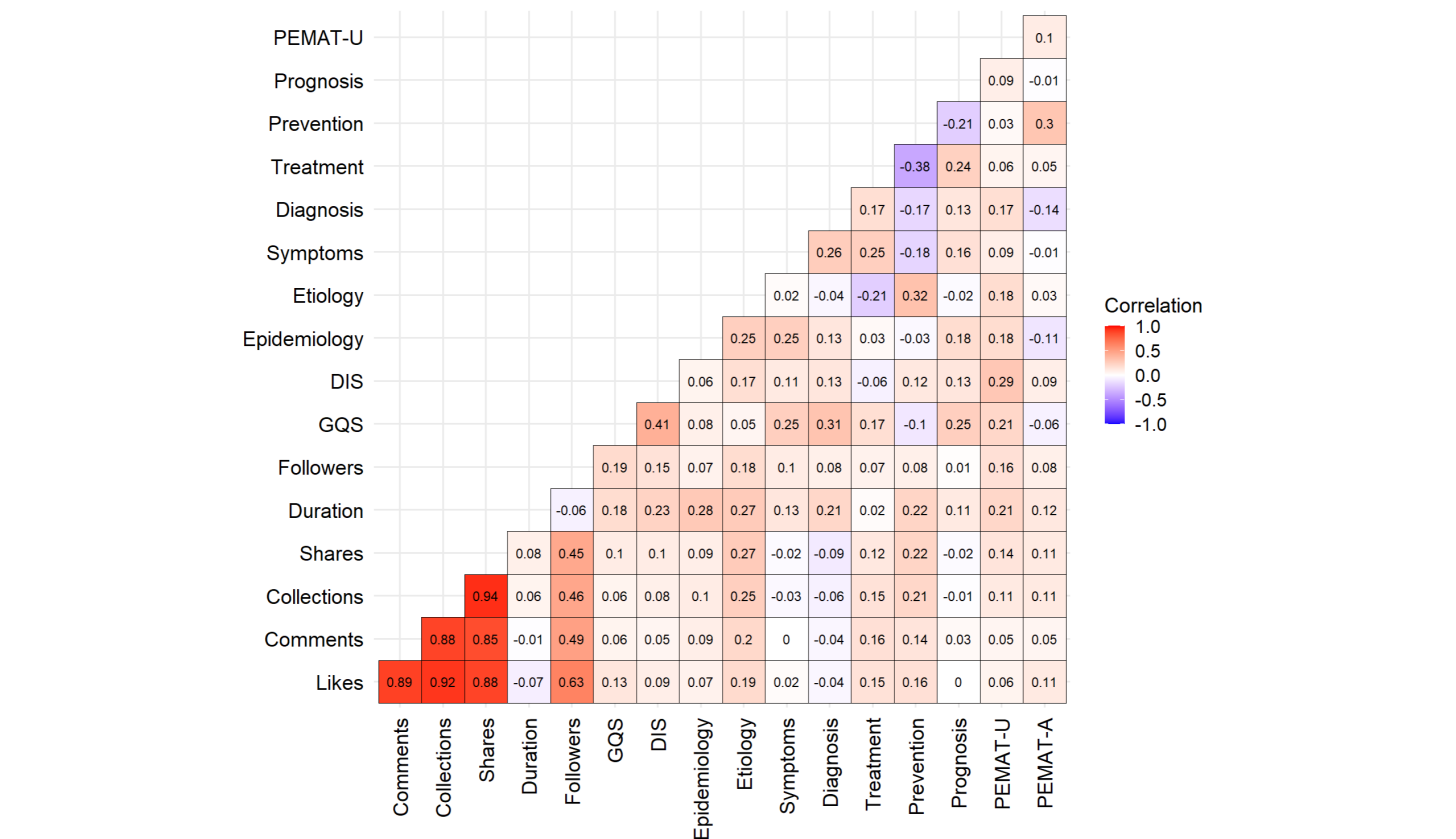
Correlation matrix of variables assessing the characteristics, quality, understandability, and actionability of uterine fibroid videos. DIS:mDISCERN; GQS: Global Quality Score; PEMAT-A: Patient Education Materials Assessment Tool-Actionability; PEMAT-U: Patient Education Materials Assessment Tool-Understandability.

Spearman rank correlation analysis was used to assess the relationships among quality scores, interaction data, and video content, as shown in Figure 7. The results indicate strong positive correlations between likes and comments, favorites (0.88<*r*<0.94), and shares, while follower count exhibits moderate to strong positive correlations with interaction data (0.45<*r*<0.63). The correlation between GQS and mDISCERN scores is *r*=0.41, with a significance level of *P*<.01, suggesting a moderate positive relationship between the two. This finding implies that higher quality scores may be associated with improved content distribution characteristics. Etiology demonstrates weak positive correlations (0.19<*r*<0.27) with interaction data (likes, comments, favorites, and shares), indicating that viewers may have a heightened interest in etiological content. Video interaction metrics exhibit only a slight positive correlation with both GQS and mDISCERN scores (0.05<*r*<0.10).

Video understandability (PEMAT-U) shows weak positive correlations with quality (GQS) and reliability (mDISCERN), with specific *r* values of 0.21 and 0.29, suggesting that content with higher quality scores tends to exhibit greater user-friendliness, reflecting a stronger alignment between content design and user needs. The actionability of videos, as assessed by the Patient Education Materials Assessment Tool for Audiovisual Materials (PEMAT-A), shows only a moderately low positive correlation with the prevention domain (*r*=0.30), while exhibiting a weak negative correlation with diagnosis (*r*=−0.14). In addition, other negative correlations among video parameters were identified, such as those between prevention and treatment (*r*=−0.38), symptoms (*r*=−0.18), diagnosis (*r*=−0.17), and prognosis (*r*=−0.21), as well as between etiology and treatment (*r=*−0.21).

Stepwise regression analysis for GQS yielded 4 models, with each subsequent model incorporating additional predictors (Tables 3-6). The final model (model 4) demonstrated statistical significance (*F*_4_=18.346; *P*<.001), accounting for 25.9% of the variance in GQS (adjusted *R*²=0.259). This model, exhibiting the strongest predictive capacity, included “Completeness Score” (*β*=0.368; *t*_195_=5.732; *P*<.001), “Source” (*β*=–0.342; *t*_195_=−5.385; *P*<.001), “PEMAT-U” (*β*=0.219; *t*_195_=3.389; *P*<.001), and “PEMAT-A” (*β*=−0.154; *t*_195_=−2.475; *P*<.05) as predictors. The 95% CIs for the unstandardized coefficients (B) confirmed the significance of each predictor: Completeness Score (0.111-0.228), Source (−0.588 to −0.273), PEMAT-U (0.008-0.029), and PEMAT-A (−0.007 to −0.001). Tolerance values for the predictors ranged from 0.895 to 0.964, and variance inflation factor values ranged from 1.000 to 1.118, indicating the absence of multicollinearity and supporting the stability of the regression coefficients.

**Table 3.** Stepwise regression analysis predicting Global Quality Scores of uterine fibroid videos on TikTok and Bilibili.

Model	Predictors included	*R* ^2^	Adjusted *R*^2^	SE of the estimate	*F* change (*df*)	*P* value	ANOVA	
							*F* test (*df*)	*P* value
1	Constant and completeness score	0.133	0.129	0.799	30.44 (1, 198)	<.001	30.44 (1, 198)	<.001
2	Constant and completeness score and source	0.213	0.205	0.763	20.095 (1, 197)	<.001	26.735 (2, 197)	<.001
3	Constant and completeness score, source, and PEMAT-U^a^	0.251	0.239	0.747	9.709 (1, 196)	.002	21.848 (3,196)	<.001
4	Constant and completeness score, source, and PEMAT-U and PEMAT-A^b^	0.273	0.259	0.737	6.217 (1, 195)	.01	18.346 (4,195)	<.001

a PEMAT-U: Patient Education Materials Assessment Tool-Understandability.

b PEMAT-A: Patient Education Materials Assessment Tool-Actionability.

**Table 4.** Stepwise regression coefficients, statistical significance, and collinearity assessment for the prediction of Global Quality Scores of uterine fibroid videos.

Model and predictors		Unstandardized coefficients		Standardized coefficients	*t* test (*df*)	*P* value	Collinearity statistics	
		B (95% CI)	SE	β			Tolerance	VIF^a^
1								
Constant		2.554 (2.238 to 2.771)	0.110	—^b^	23.228 (198)	<.001	—	—
Completeness score		0.168 (0.108 to 0.228)	0.030	0.365	5.517 (198)	<.001	1.000	1.000
2								
Constant		2.992 (2.709 to 3.275)	0.143	—	20.867 (197)	<.001	—	—
Completeness score		0.191 (0.133 to 0.249)	0.030	0.415	6.462 (197)	<.001	.970	1.031
Source		–0.362 (–0.521 to –0.203)	0.081	–0.288	–4.483 (197)	<.001	0.970	1.031
3								
Constant		1.841 (1.062 to 2.620)	0.395		4.661 (196)	<.001	0.906	1.103
Completeness score		0.167 (0.108 to 0.226)	0.030	0.363	5.582 (196)	<.001	0.952	1.051
Source		–0.396 (–0.553 to –0.239)	0.080	–0.315	-4.969 (196)	<.001	0.904	1.106
PEMAT-U		0.017 (0.006 to 0.028)	0.005	0.203	3.116 (196)	.002	—	—
4								
Constant		1.982 (1.204 to 2.579)	0.394	—	5.028 (195)	<.001	0.905	1.104
Completeness score		0.169 (0.111 to 0.228)	0.030	0.368	5.732 (195)	<.001	0.923	1.083
Source		–0.430 (–0.588 to –0.273)	0.080	–0.342	–5.385 (195)	<.001	0.895	1.118
PEMAT-U^c^		0.018 (0.008 to 0.029)	0.005	0.219	3.389 (195)	<.001	0.964	1.038
PEMAT-A^d^		–0.004 (–0.007 to –0.001)	0.002	–0.154	–2.475 (195)	.01	—	—

a VIF: variance inflation factor.

b Not applicable.

c PEMAT-U: Patient Education Materials Assessment Tool-Understandability.

d PEMAT-A: Patient Education Materials Assessment Tool-Actionability.

**Table 5.** Stepwise regression analysis predicting modified DISCERN scores of uterine fibroid videos on TikTok and Bilibili.

Model	Predictors included	*R* ^2^	Adjusted *R*^2^	SE of the estimate	F change (*df*)	*P* value	ANOVA	
							F test (*df*)	*P* value
1	Constant and PEMAT-U^a^	0.09	0.086	0.469	19.648 (1, 198)	<.001	19.648 (1, 198)	<.001
2	Constant, PEMAT-U, and completeness score	0.113	0.104	0.464	5.077 (1, 197)	.03	12.565 (2, 197)	<.001
3	Constant, PEMAT-U, completeness score and treatment	0.131	0.118	0.461	4.019 (1, 196)	.05	9.844 (3, 196)	<.001
4	Constant, PEMAT-U, completeness score, treatment, and epidemiology	0.152	0.135	0.456	4.86 (1, 195)	.03	8.744 (4, 195)	<.001

a PEMAT-U: Patient Education Materials Assessment Tool-Understandability.

**Table 6.** Stepwise regression coefficients, statistical significance, and collinearity assessment for the prediction of modified DISCERN scores of uterine fibroid videos.

Model (Predictors)	Unstandardized coefficients		Standardized coefficients	*t* (*df*)	*P* value	Collinearity statistics	
	B (95% CI)	SE	β			Tolerance	VIF^a^
1							
Constant	0.993 (0.508 to 1.479)	0.246	—^b^	4.035 (198)	<.001		—
PEMAT-U^c^	0.014 (0.008 to 0.021)	0.003	0.300	4.433 (198)	<.001	1.000	1.000
2							
Constant	1.023 (0.542 to 1.505)	0.244	—	4.193 (197)	<.001	—	
PEMAT-U	0.012 (0.006 to 0.019)	0.003	0.256	3.669 (197)	<.001	0.922	1.085
Completeness score	0.042 (0.005 to 0.078)	0.018	0.157	2.253 (197)	.03	0.922	1.085
3							
Constant	1.084 (0.603 to 1.566)	0.244	—	4.442 (196)	<.001	—	—
PEMAT-U	0.012 (0.006 to 0.019)	0.003	0.252	3.632 (196)	<.001	0.921	1.086
Completeness score	0.058 (0.019 to 0.098)	0.020	0.221	2.897 (196)	.004	0.763	1.311
Treatment	–0.124 (–0.246 to –0.002)	0.062	–0.147	–2.005 (196)	.04	0.821	1.218
4							
Constant	1.025 (0.545 to 1.505)	0.243	—	4.213 (195)	<.001	—	
PEMAT-U	0.013 (0.006 to 0.019)	0.003	0.260	3.781 (195)	<.001	0.918	1.089
Completeness score	0.087 (0.040 to 0.134)	0.024	0.330	3.655 (195)	<.001	0.534	1.872
Treatment	–0.154 (–0.277 to –0.030)	0.063	–0.183	–2.453 (195)	.02	0.783	1.278
Epidemiology	–0.221 (–0.419 to –0.023)	0.100	–0.177	–2.205 (195)	0.03	0.671	1.491

a VIF: variance inflation factor.

b Not applicable.

c PEMAT-U: Patient Education Materials Assessment Tool-Understandability.

Similarly, stepwise regression analysis for mDISCERN generated 4 models, with each building upon the previous by adding more predictors (Tables5  6). The final model (model 4) was statistically significant (*F*_4_=8.744; *P*<.001) and explained 13.5% of the variance in mDISCERN scores (Adjusted *R*²=0.135). This model, which showed the greatest predictive power, incorporated PEMAT-U (*β*=0.260; *t*_195_=3.781; *P*<.001), “Completeness Score” (*β*=0.330; *t*_195_=3.655; *P*<.001), “Treatment” (*β*=−0.183; *t*_195_=−2.453; *P*<.05), and “Epidemiology” (*β*=−0.177; *t*_195_=−2.205; *P*<.05) as predictors. The 95% CIs for the unstandardized coefficients (B) corroborated the significance of each predictor: PEMAT-U (0.006-0.019), Completeness Score (0.040-0.134), Treatment (−0.277 to −0.030), and Epidemiology (−0.419 to −0.023). Variance inflation factor values for all models remained below 2, confirming the absence of significant multicollinearity. PEMAT-U and “Completeness Score” positively influenced mDISCERN scores, while “Treatment” and “Epidemiology” exhibited a negative association.

## Discussion

### Principal Findings

This study reveals that the quality of health-related content on uterine fibroids across short video platforms is generally inadequate, aligning with previous research on liver cancer [15]. No significant quality differences were observed between TikTok and Bilibili, although TikTok showed a slight edge, particularly in videos produced by experts, which tended to be of higher quality.

During data collection, Bilibili excluded numerous in-depth instructional videos on surgery, pathology, and medical examinations, likely due to their specialized focus on medical professionals rather than the general public. In contrast, TikTok lacked any comparable content, suggesting Bilibili’s greater propensity for hosting academically oriented, in-depth medical resources. However, platform policies may prioritize broader appeal over highly specialized educational material. For platforms, striking a balance between commercial attractiveness and educational value is crucial for fostering a healthier, more accurate, and sustainable content ecosystem.

An interesting pattern emerged when comparing the sources of videos on both platforms. On TikTok, the majority of video creators were professionals with medical expertise, such as gynecologists, radiologists, oncologists, and practitioners of traditional Chinese medicine who also engage in modern health care. The only exception was a fitness blogger who posted a video about uterine fibroids, but this content was excluded from the analysis due to its relatively low overall ranking. In contrast, Bilibili featured a broader range of video creators, including not only medical professionals but also a small number of medical institutions, nonmedical organizations, and individuals without formal medical backgrounds. This difference in content creators reflects, in part, the distinct demographic profiles of the 2 platforms’ audiences.

The completeness scores of video content are generally low, typically below 7 points, and show a moderate positive correlation with the length of short videos. Creating high-quality content that is both thematically relevant and logically coherent within a brief time frame has become a key area of focus for content creators. The advantages of professional individuals and institutions, stemming from their resources, experience, and expertise, likely contribute to their better performance in this area. In contrast, nonprofessional creators tend to show more variation in their results, potentially influenced by factors such as their knowledge base, available resources, and level of training. Future research could investigate how these variables affect the performance of different groups in producing such content, providing more insightful guidance for practice and policy development in related domains.

### Correlation and Stepwise Regression Analysis of Video Quality, Reliability, and Video Characteristics

The weak correlation observed between video interaction metrics and GQS/mDISCERN scores suggests that audience engagement, while significant, is not a reliable indicator of video content quality and reliability. This finding corroborates the results reported by Cui et al [31], but diverges from those of Li et al [32] concerning osteoporosis. These discrepancies highlight the need for enhanced public discernment in differentiating between high-quality and low-quality video resources.

The primary preventive measures for uterine fibroids include regular check-ups (for early detection and intervention), weight management, avoidance of prolonged exogenous estrogen exposure, a healthy diet (eg, reducing red meat intake), and chronic disease management. These preventive measures are relatively feasible for the general public, thereby accounting for the positive correlation between video actionability and prevention. In contrast, diagnosis requires specialized medical knowledge, rendering it less practicable for the general public and thus explaining the observed negative correlation.

The stepwise regression analysis revealed a significant positive predictive relationship between “Information Completeness Score” and GQS scale scores, while “Video Source” exhibited a negative association. Specifically, videos originating from nonprofessionals tended to receive lower GQS ratings from viewers. This could be attributed to potential limitations in the accuracy, professional rigor, and presentation quality of content from nonprofessional sources compared to that from professionals and specialized medical institutions, potentially failing to meet viewers’ demands for high-quality information and consequently leading to lower evaluations. Furthermore, even videos from professional channels might receive lower GQS ratings from viewers with nonspecialized backgrounds if the content is overly technical and uses obscure terminology. Future research could further investigate the specific content characteristics of videos from different sources and their impact on viewers’ GQS evaluations. Conversely, the PEMAT-U scale and Information Completeness Score positively predicted mDISCERN scores, whereas variables related to “Treatment” and “Epidemiology” showed negative associations. It is crucial to interpret the negative coefficients between variables and scores with caution, as these results do not necessarily imply causation.

### Practical Significance

Short videos, known for their conciseness, effective visual communication, ease of production, and high interactivity, address the varied preferences of different audience groups. To stand out in a highly competitive environment, both individual creators and corporate organizations must focus on the 3 key principles: “visual appeal, emotional connection, and actionable guidance.” However, in response to market pressures, clickbait titles have become increasingly common, often paired with content that is shallow or misleading. This trend highlights the urgent need to improve the overall quality of video production.

Globally, TikTok has emerged as the fastest-growing social media platform among children and young people [33], with substantial potential as a public health tool [34]. Personal health advice disseminated via social media appears to exert a profound influence on adolescents [35], and misinformation in some videos can rapidly proliferate, adversely affecting public health [36]. A study on tonsil stones revealed that children attempting untested remedies from videos suffered significant harm [35].

Platform regulation and expert engagement are crucial for enhancing the quality of online health information. Based on the Elaboration Likelihood Model (ELM), the Heuristic-Systematic Model (HSM), and the Media Trust Model, the following actionable recommendations are proposed: (1) Establish a “Health Video Tagging Mechanism”: implement a standardized system enabling creators to categorize health-related videos with specific tags (eg, symptoms, treatment, and prevention) and indicate the level of evidence or source type (eg, expert opinion, personal experience, and research-based); (2) enhance expert verification methods: implement more rigorous and standardized procedures, requiring proof of licensure and establishing a transparent process for users to report potentially misleading or unqualified “expert” accounts; (3) prioritize verified expert content algorithmically: content originating from accounts confirmed as “experts” should receive preferential algorithmic weighting to increase its visibility and reach; this directly addresses the need for expert involvement and leverages platform algorithms to promote credible information dissemination; and (4) promote evidence-based content structures: provide creators with guidelines and templates for structuring their videos to clearly and concisely emphasize essential, evidence-based information. Encourage the use of interactive features (eg, quizzes and polls) to enhance engagement while reinforcing accurate information.

### Strengths and Limitations

This research scrutinized video material sourced from 2 major Chinese short-form video applications, covering a wide spectrum of age groups. Qualified health care experts appraised the videos, judging their quality, reliability, understandability, and actionability through the usage of the GQS, mDISCERN, and PEMAT-A/V rating methodologies. To strengthen the robustness of the research, Spearman’s rank correlation coefficient and stepwise regression techniques were implemented. The outcomes present valuable direction for developers of content endeavoring to improve the standard of health-related information sharing.

Nonetheless, several constraints merit attention. First, the confinement to videos in the Chinese language restricts the extent to which the results can be broadly applied. Subsequent studies ought to delve into how well the sample reflects the overall population and the restrictions imposed by choosing a specific language and platform. Second, choosing the top 100 most popular videos could potentially introduce bias caused by algorithms. Third, the appropriateness of using the GQS and mDISCERN instruments for evaluating video content demands further confirmation. Finally, the restricted number of videos from both professional and nonprofessional institutions may not adequately depict the wider range of videos available. Subsequent investigations should strive to enlarge the number of samples within these classifications.

While the selection of the top 100 videos via a comprehensive ranking intended to identify prevalent content, it is crucial to recognize the potential impact of platform-specific algorithms on video visibility and ranking. TikTok’s algorithm, known to favor concise and visually stimulating content, may have elevated videos exhibiting these attributes in the top ranks, potentially leading to an underrepresentation of lengthier, more in-depth explanations. Similarly, Bilibili’s algorithm, with its emphasis on community engagement and potentially longer-form content, might have highlighted a distinct set of videos. These inherent algorithmic biases could affect the generalizability of our findings to the entirety of uterine fibroid information present on these platforms and may have influenced the representativeness of the analyzed content. Subsequent research using alternative sampling strategies is necessary to achieve a more holistic understanding of the online information landscape concerning uterine fibroids.

### Conclusion

This research evaluated the quality of health-related information about uterine fibroids on the TikTok and Bilibili platforms, revealing that the overall quality is generally inadequate, with minimal differences between the 2 platforms. However, TikTok videos tend to show slightly higher quality compared to those on Bilibili, while content created by medical experts stands out with significantly better quality. The growing availability of online educational resources has highlighted increasing disparities in content quality and professionalism across platforms. While Bilibili hosts a higher percentage of videos with an academic focus, users must still apply discernment to ensure the information they encounter is accurate. Future studies could investigate strategies to improve video quality on these platforms and enhance users’ ability to critically assess and use the available content. Furthermore, research may explore ways to balance professionalism and accessibility to better meet the diverse needs of various audiences. It is therefore recommended that platforms strengthen oversight and encourage more active involvement from medical professionals in content production. In addition, medical experts should address common misconceptions and inaccuracies in health-related videos, ensuring that the public receives more reliable and scientifically sound health information.

## Supplementary material

10.2196/75120 Multimedia Appendix 1 Uterine Fibroid dataset.

10.2196/75120 Multimedia Appendix 2 Evaluation of uterine fibroid–related videos on TikTok and Bilibili by quality, engagement, understandability, actionability, and content completeness across sources and content categories

10.2196/75120 Multimedia Appendix 3 Uterine Fibroid Completeness Scoring Table.

## Abbreviations

ELM: Elaboration Likelihood Model
GQS: Global Quality Score
HSM: Heuristic-Systematic Model
mDISCERN: modified DISCERN
PEMAT: Patient Education Materials Assessment Tool
PEMAT-A: Patient Education Materials Assessment Tool-Actionability
PEMAT-A/V: Patient Education Materials Assessment Tool for Audio/Visual Materials
PEMAT-U: Patient Education Materials Assessment Tool-Understandability
