# Supplementary material for: Quality and Dissemination of Uterine Fibroid Health Information on TikTok and Bilibili: Cross-Sectional Study
Source: JMIR Form Res. 2025 Aug 1;9:e75120. doi: 10.2196/75120 (PMC12316441; doi:10.2196/75120)
Supplement: Multimedia Appendix 2 [file formative-v9-e75120-s002.docx]

| Table S1**.** Description of the global quality score(5-point scale)for evaluating the quality of the videos with uterine fibroids information. | |
| --- | --- |
| Score | Description |
| 1 | Poor quality,poor flow of the site,most information missing,and it is useless for patients. |
| 2 | Generally poor quality and flow,the content logic is poor, although some information is listed, more important information  is still missing, and the use of patients is very limited. |
| 3 | Moderate quality,suboptimal flow,some important information is adequately discussed, somewhat useful for patients. |
| 4 | Good quality and flow,the video logic is clear and smooth,most of the relevant information is listed,useful for patients. |
| 5 | Excellent quality and flow,the video logic is clear, and the content is very smooth, very useful for patients. |

| Table S2. Distribution of characteristics of uterine fibroid videos across different sources on TikTok and Bilibili. | | | | | | | | | |
| --- | --- | --- | --- | --- | --- | --- | --- | --- | --- |
| Variable median (IQR) | Professional individuals | | | Nonprofessional individuals | | Professional institutions | | Nonprofessional institutions | |
|  | TikTok (n=83) | | Bilibili (n=52) | TikTok (n=16) | Bilibili (n=39) | TikTok (n=1) e | Bilibili (n=3) | TikTok (n=0) | Bilibili (n=6) |
| Likes | 445(238-1377) | | 30(7-109) | 434(264-927) | 53 (9-560) | 386 | 16(13-338) |  | 74(39-158) |
| Comments | | 27(11-75) | 3(0-14) | 139(43-322) | 6(0-92) | 288 | 2(1-28) |  | 4(0-32) |
| Collections | 119(65-354) | | 16(3-72) | 204(98-588) | 69(8-331) | 68 | 10(7-350) |  | 36(19-127) |
| Shares | 63(29-224) | | 13(2-66) | 90(56-364) | 54(2-333) | 204 | 29(4-240) |  | 9(4-82) |
| Duration | 65(46-91) | | 108(67-166) | 76(56-95) | 219(98-397) | 41 | 117(100-564) |  | 295(286-393) |
| Followers | 116000 (30000-696000) | | 14000 (408-18000) | 89500 (33250-365500) | 6369 (1200-47000) | 16000 | 2580 (261-73000) |  | 191000 (161000-191000) |
| GQSa | 3(3-4) | | 3(3-4) | 2(2-3) | 2(2-3) | 2 | 3(3-3) |  | 3(3-4) |
| mDISb | 2(2-2) | | 2(2-2) | 2(2-2) | 2(2-2) | 2 | 3(2-3) |  | 2(2-3) |
| PEMAT-Uc | 77% (69%-83%) | | 77% (69%-83%) | 76% (69%-85%) | 69% (62%-77%) | 85% | 77% (69%-77%) |  | 92% (83%-92%) |
| PEMAT-Ad | 67% (33%-67%) | | 67% (33%-67%) | 33% (33%-67%) | 67% (33%-67%) | 67% | 75% (0-75%) |  | 0 (0-33%) |

^a^GQS: Global Quality Score.

^b^mDIS: modified DISCERN.

^c^PEMAT-U: Patient Education Materials Assessment Tool-Understandability score.

^d^PEMAT-A: Patient Education Materials Assessment Tool-Actionability score.

^e^Among professional institutions, TikTok (n=1) presents single data points in the corresponding column, which therefore represent individual values rather than medians or interquartile ranges (IQR).

| Table S3.Distribution of characteristics of uterine fibroid videos across different content categories on TikTok. | | | | | |
| --- | --- | --- | --- | --- | --- |
| Variable（TikTok） median (IQR) | Disease knowledge （n=31） | Etiology (n=5) | Symptoms （n=5） | Treatment （n=48） | Prevention and Prognosis （n=11） |
| Likes | 544 （272-1908） | 337 （251-9291） | 245 （108-417） | 412 （214-689） | 721 （226-105000） |
| Comments | 27 （13-288） | 41 （22-757） | 4 （4-13） | 35 （18-92） | 51 （1-5947） |
| Collections | 131 （58-509） | 177 （57-14659） | 40 （20-92） | 122 （71-354） | 276 （55-52000） |
| Shares | 72 （29-476） | 258 （96-4603） | 19 （9-59） | 58 （36-147） | 162 （32-46000） |
| Duration | 77 （59-100） | 54 （48-87） | 54 （43-63） | 57 （45-81） | 75 （33-481） |
| Followers | 184000 （23000-864000） | 228000 （58000-1733000） | 419000 （30000-617500） | 75000 （16000-170500） | 1327000 （1933-6486000） |
| GQS^a^ | 4（3-4） | 2（2-3） | 3（2.5-4.5） | 3（2-4） | 3（2-4） |
| mDIS^b^ | 2（2-2） | 2（2-2） | 2（2-2.5） | 2（2-2） | 2（1-3） |
| PEMAT-U^c^ | 77% （69%-77%） | 83% （72%-88%） | 85% （74%-89%） | 69% （67%-77%） | 83% （54%-92%） |
| PEMAT-A^d^ | 67% （33%-67%） | 33% （17%-67%） | 67% （33%-100%） | 67% （33%-67%） | 67% （33%-100%） |

^a^GQS: Global Quality Score.

^b^mDIS: modified DISCERN.

^c^PEMAT-U: Patient Education Materials Assessment Tool-Understandability score.

^d^PEMAT-A: Patient Education Materials Assessment Tool-Actionability score.

| Table S4. Distribution of characteristics of uterine fibroid videos across different content categories on Bilibili. | | | | | |
| --- | --- | --- | --- | --- | --- |
| Variable（Bilibili） median (IQR) | Disease knowledge （n=32） | Etiology (n=3) | Symptoms （n=4） | Treatment （n=31） | Prevention and Prognosis （n=30） |
| Likes | 113 （11-833） | 8661 （68-8661） | 2 （1-37） | 31 （7-80） | 37.5 （12-233） |
| Comments | 8 （0-101） | 297 （9-479） | 0 （0-3） | 3 （0-9） | 3.5 （1-24） |
| Collections | 85 （9-345） | 607 （46-2077） | 2.5 （0-17） | 15 （3-63） | 32.5 （6.25-141） |
| Shares | 44 （7-322） | 1170 （4-8003） | 1.5 （0-12） | 9 （1-61） | 20 （4-102） |
| Duration | 222 （123-529） | 297 （145-373） | 93 （58-139） | 128 （68-243） | 102.5 （72-176） |
| Followers | 18444 （817-171750） | 226000 （191000-2137000） | 184.5 （18-18000） | 14000 （2252-18000） | 7129 （1673-23000） |
| GQS^a^ | 3（2-4） | 4（3-4） | 3.5（2-4） | 3（3-4） | 3（2-3） |
| mDIS^b^ | 2（2-2） | 3（3-3） | 2（2-2） | 2（2-2） | 2（2-2） |
| PEMAT-U^c^ | 77% （71%-85%） | 92% （77%-92%） | 69% （69%-85%） | 77% （62%-85%） | 69% （62%-77%） |
| PEMAT-A^d^ | 33% （0-67%） | 33% （0-33%） | 50% （8%-67%） | 67% （0-67%） | 67% （67%-100%） |

^a^GQS: Global Quality Score.

^b^mDIS: modified DISCERN.

^c^PEMAT-U: Patient Education Materials Assessment Tool-Understandability score.

^d^PEMAT-A: Patient Education Materials Assessment Tool-Actionability score.

| Table S5. Descriptive statistics of completeness scores for uterine fibroid video content. | | | | | | | | | |
| --- | --- | --- | --- | --- | --- | --- | --- | --- | --- |
| Video content,n(%) | Not involve (0 points) | | | Partial explanation (1 point) | | | Full explanation (2 points) | | |
|  | TikTok | Bilibili | Total | TikTok | Bilibili | Total | TikTok | Bilibili | Total |
| Epidemiology | 88(88) | 82(82) | 170(85) | 11(11) | 17(17) | 28(14) | 1(1) | 1(1) | 2(1) |
| Etiology | 70(70) | 69(69) | 139(69.5) | 27(27) | 21(21) | 48(24) | 3(3) | 10(10) | 13(6.5) |
| Symptoms | 49(49) | 53(53) | 102(51) | 21(21) | 23(23) | 44(22) | 30(30) | 24(24) | 54(27) |
| Diagnosis | 70(70) | 66(66) | 136(68) | 29(29) | 30(30) | 59(29.5) | 1(1) | 4(4) | 5(2.5) |
| Treatment | 23(23) | 37(37) | 60(30) | 66(66) | 57(57) | 123(61.5) | 11(11) | 6(6) | 17(8.5) |
| Prevention | 81(81) | 66(66) | 147(73.5) | 19(19) | 34(34) | 53(26.5) | 0(0) | 0(0) | 0(0) |
| Prognosis | 62(62) | 57(57) | 119(59.5) | 37(37) | 39(39) | 76(38) | 1(1) | 4(4) | 5(2.5) |

| Table S6. Completeness scores of uterine fibroid video content across different sources. | | | | | | | |
| --- | --- | --- | --- | --- | --- | --- | --- |
| source of videos | Epidemiology mean(SD) | Etiology mean(SD) | Symptoms mean(SD) | Diagnosis mean(SD) | Treatment mean(SD) | Prevention mean(SD) | Prognosis mean(SD) |
| Professional individuals | 0.10(0.32) | 0.27(0.54) | 0.75(0.86) | 0.35(0.51) | 0.83(0.58) | 0.21(0.41) | 0.45(0.57) |
| Nonprofessional individuals | 0.27(0.49) | 0.58(0.66) | 0.71(0.81) | 0.29(0.53) | 0.71(0.53) | 0.44(0.50) | 0.35(0.48) |
| Professional institutions | 0.25(0.50) | 0.75(0.96) | 0.75(0.96) | 0.50(1.00) | 0.50(1.00) | 0.25(0.50) | 0.25(0.50) |
| Nonprofessional institutions | 0.50(0.55) | 0.50(0.84) | 1.50(0.84) | 0.67(0.52) | 0.67(0.82) | 0.00(0.00) | 0.83(0.41) |
