# Supplementary material for: Quality and Dissemination of Uterine Fibroid Health Information on TikTok and Bilibili: Cross-Sectional Study
Source: JMIR Form Res. 2025 Aug 1;9:e75120. doi: 10.2196/75120 (PMC12316441; doi:10.2196/75120)
Supplement: Multimedia Appendix 3 [file formative-v9-e75120-s003.doc]

**Uterine Fibroid Integrity Scoring Document**

The development of this Completeness of Content Scoring Table is grounded in the "Comprehensive Review of Uterine Fibroids: Developmental Origin, Pathogenesis, and Treatment[1]" and the "Chinese Expert Consensus on Diagnosis and Treatment of Uterine Fibroids.[2]"

This was reviewed and consensus was reached by three gynecological experts.

1. **Epidemiology**

1) Global incidence rate (approximately 70% of women of reproductive age)

2) Incidence in China (approximately 30% of women of reproductive age)

3) High-risk age groups (30-50 years old)

4) Racial disparities (higher incidence in African American women)

2. **Etiology**

1) Abnormal estrogen levels (dominant factor)

2) Progesterone imbalance

3) Genetic predisposition (family history)

4) Abnormal growth factors (e.g., TGF-β)

5) Race and genetic polymorphisms

3. **Symptoms**

1) Menstrual abnormalities (increased menstrual flow, prolonged periods)

2) Pelvic pressure or pain

3) Urinary frequency or constipation (due to compression of bladder/rectum)

4) Infertility or recurrent miscarriage (submucosal fibroids)

5) Abdominal mass (large fibroids)

4. **Diagnosis**

1) Gynecological palpation (enlarged, irregularly shaped uterus)

2) Ultrasound (transvaginal/abdominal)

3) Magnetic resonance imaging (MRI, precise localization)

4) Hysteroscopy (submucosal fibroids)

5) Laboratory tests (exclude anemia, assess hormone levels)

5. **Treatment**

1) Medical therapy: Gonadotropin-releasing hormone agonists (GnRH-a), oral contraceptives (control bleeding), mifepristone (reduce fibroid size)

2) Surgical therapy: Hysteroscopic/laparoscopic minimally invasive surgery, myomectomy (preserve uterus), hysterectomy (for women without fertility needs)

3) Interventional therapy: Uterine artery embolization (UAE), ablation

4) Observation(asymptomatic small fibroids)

6. **Prevention**

1) Regular gynecological examinations (early detection and intervention)

2) Weight control (obesity increases estrogen levels)

3) Avoid long-term exogenous estrogen (e.g., hormone replacement therapy)

4) Healthy diet (reduce red meat, increase vegetable intake)

5) Manage chronic diseases (e.g., hypertension, diabetes)

7. **Prognosis**

1) Benign tumors, extremely low malignancy risk (<0.5%)

2) Recurrence risk post-treatment (approximately 20%-30%)

3) Management of pregnancy with fibroids (requires individualized assessment)

4) Natural regression after menopause (due to decreased estrogen)

**Scoring Instructions**: Each section is scored 0-2 points. The scoring rule for sub-items is as follows: zero points for no mentions, one point for mentioning one or two sub-items (1-2), and two points for mentioning three or more sub-items (≥3). Total score: 14 points.

**Scoring Example:** Based on Video Content.

| Epidemiology | Etiology | Symptoms | Diagnosis | Treatment | Prevention | Prognosis | Completeness score |
| --- | --- | --- | --- | --- | --- | --- | --- |
| 0 | 1 | 0 | 0 | 1 | 0 | 1 | 3 |
| 2 | 2 | 2 | 1 | 2 | 1 | 1 | 11 |

**References:**

1. Yang Q, Ciebiera M, Bariani MV, Ali M, Elkafas H, Boyer TG, et al. Comprehensive Review of Uterine Fibroids: Developmental Origin, Pathogenesis, and Treatment. Endocr Rev. 2022 Aug 1;43(4):678–719.

2. Chinese Expert Consensus Panel on Diagnosis and Treatment of Uterine Fibroids. Consensus for diagnosis and treatment of uterine myoma. Chinese Journal of Obstetrics and Gynecology. 2017 Dec 25;52(12):793–800.
